# Cross-Fostering Implications for Pig Mortality, Welfare and Performance

**DOI:** 10.3389/fvets.2018.00123

**Published:** 2018-06-06

**Authors:** Julia A. Calderón Díaz, Edgar García Manzanilla, Alessia Diana, Laura A. Boyle

**Affiliations:** ^1^Pig Development Department, Teagasc Animal and Grassland Research and Innovation Centre, Fermoy, Ireland; ^2^School of Veterinary Medicine, University College Dublin, Dublin, Ireland

**Keywords:** carcass traits, cross-fostering, health, mortality, growth performance, pigs, welfare

## Abstract

This study aimed to (1) identify cross-fostering (CF) practices employed on a commercial farm; (2) characterize CF pigs according to litter of origin traits, and (3) investigate the implications of CF practices on pig mortality, performance and welfare. This was an observational study whereby pigs were managed according to normal farming practice. Pigs (*n* = 1,016) born within 1 week were classified as non-CF (NCF); CF during the first (CFW1) and second or third (CFW2+) weeks of lactation. Pigs were individually weighed and inspected for the presence of tail (TL), ear (EL) and body lesions (BL) at weaning (7.03 ± 1.61 kg) and at the end of the first (12.9 ± 3.03 kg) and second (31.9 ± 5.50 kg) weaner and grower (66.3 ± 9.12 kg) stages. Mortality was recorded through to slaughter (c. 115 kg). At slaughter, TL were scored and carcass characteristics, presence of pleurisy, enzootic pneumonia, pericarditis and heart condemnations were recorded. 40.8% of CF pigs were CFW1; ANOVA tests revealed these were born to sows with a higher number of piglets born alive than NCF pigs (14.6 ± 2.61 and 12.8 ± 2.68, respectively). The remaining 59.2% of CF pigs were CFW2+; these were, on average, 0.14 kg lighter at birth than NCF pigs. Therefore, a nested case control design was retrospectively applied whereby pigs with complete records to slaughter, were matched for these variables to investigate associations between CF weeks, welfare and performance traits. Growth performance did not differ between CF week (*P* > 0.05); however, CFW2+ carcasses were 4.9 kg lighter (*P* < 0.05) compared with NCF and CFW1 pigs. EL were more likely in CFW1 compared to NCF and CFW2+ (*P* < 0.05) pigs. To investigate the effect of CF week on the risk of mortality, all 1,016 pigs were used. CF pigs were at higher risk of death (*P* < 0.05) with similar odds in CFW1 and CFW2+ pigs compared with NCF pigs, although other underlying factors could contribute to this result. Performance and health traits were similar between CF weeks. Early cross-fostering appeared to influence the presence of ear lesions but the mechanism is likely indirect and difficult to explain.

## Introduction

Cross-fostering (CF) is a management technique used in up to 98% of commercial pig farms (1) to increase piglet survival and to create litters with more uniform body weight (BW) (2). It is recommended that CF is kept to a minimum as it can be stressful for sows and piglets (3). Furthermore, if CF is required it should be performed as early as possible (i.e., 12–24 h after farrowing) as the teat order is not established at this time (4). Early CF also ensures maximal colostrum intake from the piglets own dam (5). CF practiced in this way can reduce pre-weaning mortality; it does not negatively affect growth performance and may not affect piglet behavior as the CF animals adapt to their new environment relatively easily (5–7). However, recent studies report associations between CF and the presence of tail lesions (8) and a greater risk of disease such as pericarditis and greater risk of heart condemnations at slaughter (9). A possible explanation for the latter is that CF piglets might not spend enough time with their dams to consume enough colostrum to acquire immunity for protection against disease (10).

In practice, CF often continues through lactation such that piglets may be subjected to late CF (i.e., >7 days after farrowing). In fact, results from a recent survey conducted on 79 Irish pig farms show that in 51.9% of farms CF takes place 4 days after farrowing and in 46% of them only late CF is practiced (unpublished data). Van Erp-Van Der Kooij et al. (11) reported no negative consequences of CF up to 5 days after farrowing on performance indicators such as average daily gain or carcass composition. However, there is limited research about CF practices under commercial conditions and the implications for pig performance, health and welfare traits.

Late CF means that piglets are introduced to litters in which the teat order is already established which could be stressful and have a detrimental effect on survival, growth performance and behavior of both CF and resident piglets (5, 12, 13). Late CF piglets are also less likely to be present at milk letdown and they show signs of distress (7, 13). The latter is evidenced by more wandering around the pen, frequent vocalizations and performance of escape attempts (7, 12, 13). Further, late CF increases fighting (13) and the greater number of face and body scratches in such pigs suggest that they are the receivers of aggression (5). Late CF also seems to impair growth performance as late CF piglets have lower BW gains than non-CF pigs (5, 12, 14, 15). However, none of these studies followed piglets through to slaughter and the possible long term effects of late CF are unknown.

Thefore, the objectives of this study were to (1) identify CF practices employed on a commercial pig farm; (2) characterize CF pigs according to litter of origin traits, and (3) investigate the possible implications of such CF practices on pig mortality, growth performance, carcass traits and welfare and health indicators.

## Materials and methods

### Animals and housing

The study was conducted on a 1,500 Large White × Landrace sow farrow-to-finish commercial farm in Ireland. This was an observational study whereby pigs were managed as per usual practice on the farm. Details regarding animal management and measurements recorded were described previously (9). In brief, the farm followed a 1 week batch farrowing system with approximately 80 sows farrowing per week. All piglets (*n* = 1,016) born from the same weekly batch were individually tagged at birth and followed to slaughter. Pigs were tail-docked, and tooth clipped within 24 h after farrowing. Males were not castrated as per common practice in Irish pig farms. Sex, number of piglets born alive (NBA), sow parity and the stage of lactation when pigs were CF was recorded.

Pigs were classified according to the stage of lactation when they were CF as (1) non-CF (NCF; *n* = 712); (2) CF during the first (CFW1; *n* = 124), and (3) second or third (CFW2+; *n* = 180) weeks of lactation. This farm declared that it followed an all-in/all-out policy whereby pigs should spend 8 weeks in the weaner stage (4 weeks in the first and 4 weeks in the second weaner stages) after weaning, 4 weeks in the growing stage and 8 weeks in the finisher stage.

In this study, pigs were housed in mixed sex groups throughout the production cycle. Pigs were housed in groups of 55 pigs with a minimum 0.30 m^2^ per pig during the first weaner stage. Groups were split and mixed by size/BW and housed for the next 8 weeks (4 weeks in the second weaner stage and 4 weeks grower stage) in groups of 36 pigs with a minimum 0.55 m^2^ per pig. Finally, pigs were transferred to the finishing stage for 8 weeks and housed in groups of 35 with a minimum 0.65 m^2^ per pig. Wood, rubber toys and/or chains were provided as environmental enrichment. It is important to note that group composition changed between each stage according to regular farm management practice of re-grading pigs according to size/BW on transfer between each of the production stages. However, NCF, CFW1, and CFW2+ were housed together throughout the production cycle.

In each stage, rooms and pens had the same design and environmental control. Weaner and growing facilities had an automatic temperature control system with fans in the ceiling while finisher facilities had natural ventilation. In all stages, animals were housed on fully slatted floors; plastic floors for the two weaner stages and concrete floors for the grower and finisher stages. Pigs were wet-fed a common weaner diet with 18.3% CP and 10.5 MJ/DE per kg of feed; grower diet with 18.1% CP and 10.0 MJ/DE per kg of feed, and finisher diets with 16.9% CP and 9.9 MJ/DE per kg of feed. Pigs had *ad libitum* access to water via at least one nipple drinker in each pen.

### Growth performance and welfare indicators during the production cycle

Pigs were weighed individually at weaning and at the end of the first and second weaner and grower stages. Pigs were not weighed at the end of the finisher stage. Average daily gain (ADG) was calculated for each time period. At the time of weighing, pigs were inspected individually for the presence or absence of tail, ear, and body lesions by a single trained observer. Pig mortality was recorded throughout the production cycle; however, information on causes of death was not available from farm records.

### Welfare and health indicators at slaughter and carcass traits

Eight-hundred-and-twenty-four pigs reached slaughter age. All animals were slaughtered within 1 week, regardless of their BW, at 24 weeks of age. Prior to slaughter, pigs were scored for lameness by a single trained observer on a 3-point scale where 1 = non lame; 2 = mildly lame and 3 = severely lame. At slaughter, tail lesions were scored after scalding and dehairing by one trained observer as per (16). Cold carcass weight (kg), fat thickness (mm) and muscle depth (mm) were recorded by the slaughterhouse personnel using a Fat-O-Meat'er (Carometec Food Technology, Carometec A/S, Hasselunden 9, Smørum, Denmark). Percentage of lean meat was calculated according to the formula established by the European Communities Pig Carcass Grading Amendment Regulations (17):

$$\begin{array}{l} \%\text{ lean meat }=60.30-\left(0.847\times \text{fat thickness},\text{ mm}\right)\\ \;+\left(0.147\times \text{muscle},\text{ mm}\right) \end{array}$$

Pleurisy was scored using the Slaughterhouse Pleurisy Evaluation System [SPES; 18)] and enzootic pneumonia like lesions were scored according to the BPEX Pig Health Scheme (19) by one trained observer. Additionally, pericarditis and heart condemnations were recorded as present or absent as per the decision of the acting veterinary inspector on the slaughter line.

### Data management and statistical analysis

All data were analyzed in R v3.4.1 (20). For all the analyses, alpha level for determination of significance and trends were 0.05 and 0.10, respectively.

First, as this was an observational study and CF is associated with traits related to the litter of origin (9), ANOVA tests for sow parity, birth weight and NBA were conducted including data from all animals in the batch that reached slaughter (*n* = 824 pigs) to confirm that these parameters were not different between CF weeks. Statistical differences were detected for the three parameters between CF weeks. Therefore a nested case control design was retrospectively applied whereby pigs CF on different weeks were matched by sow parity, birth weight and NBA facilitating investigation between the studied traits and CF practices independently of the other underlying factors. Only animals with complete records (i.e., pigs that reached slaughter) were used for the nested case control design. The final data set for the nested case control included 62 NCF pigs, 31 CFW1 pigs CF, and 31 CFW2+ pigs. These data were used to investigate the possible implications of CF practices on growth performance, carcass traits and welfare and health indicators.

Body weight and ADG were analyzed using mixed model equations in the *lme4* package (21). CF week, production stage and their interaction were included as fixed effects. Each pig was included as a random variable. Least square means (LS means) were estimated using the *lmerTest* package (22). Multiple-comparisons as well as a Tukey adjustment for the LS means were carried out using the *multicomp* package (23).

Ear and body lesions were not observed at weaning, thus, this time point was not included in the analysis for the aforementioned lesions. As only one pig was scored as severely lame, lameness was re-classified into non-lame and lame. Tail, ear, and body lesions during the production cycle were analyzed using binomial logistic regression using the *glm* function with a binomial distribution from the *stats* package (20). Models included CF week and production stage as fixed effects. Results for logistic regression models are reported as odds ratios (OR) and their associated 95% confidence interval (CI).

Lameness prior to slaughter, carcass tail lesions, pleurisy, enzootic pneumonia, pericarditis and heart condemnations were analyzed using binomial logistic regression using the *glm* function with a binomial distribution from the *stats* package (20). Cold carcass weight, muscle and fat depth and lean meat percentage were analyzed using linear model equations using the *glm* function with a Gaussian distribution from the *stats* package (20). In all cases, models included CF week as a fixed effect. LS means were estimated using the *lsmeans* package (24).

As we were also interested in the possible associations between mortality and CF practices, mortality rates from birth to weaning and from birth to slaughter were analyzed. All 1,016 pigs were used for the mortality analyses. For both cases, two models were used. For the first one, pigs were classified according to their CF week whereas for the second model, pigs were classified as either CF, or not CF, regardless of CF week. Data were analyzed using binomial logistic regression using the *glm* function with a binomial distribution from the *stats* package (20). Models included CF week as a fixed effect.

## Results

### Cross-fostering practices and characteristics of CF pigs

**Figure 1:**
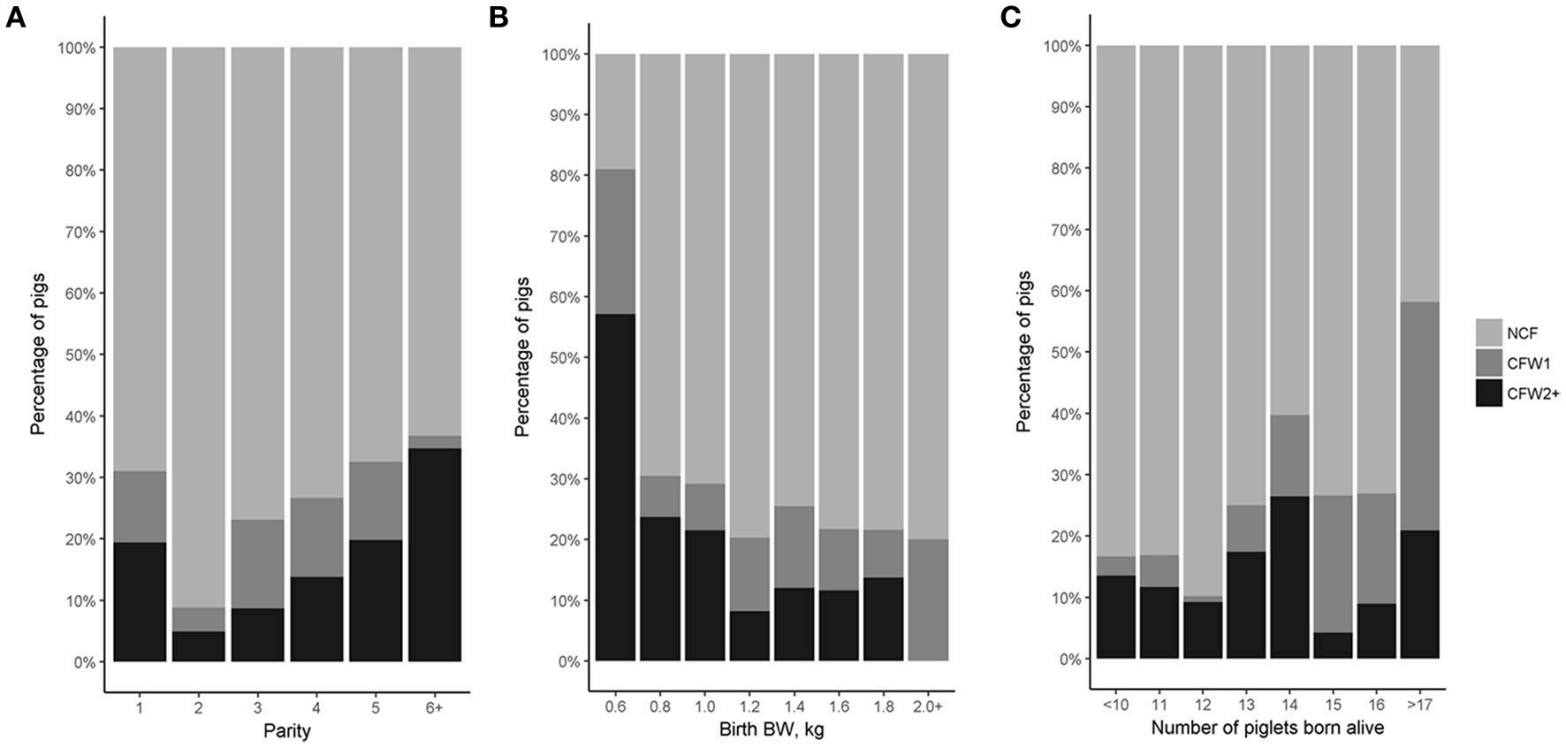
Percentage of pigs by **(A)** parity, **(B)** birth body weight (BW) and **(C)** number of piglet born alive according to cross-fostering week. One batch of pigs 1,016 born within 1 week was followed from birth to slaughter in a farrow-to-finish commercial farm. Pigs were managed as per usual practice on the farm and they were either not cross-fostered (NCF), cross-fostered during the first week of lactation (CFW1) or cross-fostered on the second or third week of lactation (CFW2+).

In total, 29.9% of piglets born alive were CF. Two CF practices were identified: (1) early CF (i.e., CF during the first week of lactation; CFW1) was performed in 40.8% of CF pigs and (2) late CF (i.e., after 1 week of lactation; CFW2+) was performed in 59.2% of CF pigs. Mean parity was higher in CFW2+ pigs (3.65 ± 1.70) compared with mean parity in NCF (3.26 ± 1.42) and CFW1 (3.33 ± 1.32; *P* < 0.05) pigs. Mean BW at birth was lower for CFW2+ (1.26 ± 0.33 kg) than for NCF (1.41 ± 0.29 kg) and CFW1 (1.39 ± 0.29 kg) pigs (*P* < 0.001; Figure 1) with 5.2% of NCF; 7.8% of CFW1 pigs and 18.2% of CFW2+ pigs having a birth BW of < 0.95 kg which was identified as the threshold for a higher risk of mortality during the production cycle in this population (9). Number of piglets born alive differed between the 3 CF weeks with lower NBA for NCF pigs (12.69 ± 2.69 piglets) than for CFW1 (14.81 ± 2.53 piglets) and CFW2+ (13.28 ± 2.62) pigs (*P* < 0.01).

### Growth performance and welfare indicators during the production cycle

One-hundred-and-ninety-two (18.9%) pigs died during the study. A total of 106 pigs (55.2%) died during the lactation period, 24 pigs died during the first and second weaner stages (12.5%), 3 pigs died during growing (1.5%), 14 pigs (7.3%) died during the finishing stage and 45 (23.4%) pigs were euthanized for a separate study investigating respiratory pathologies. From the euthanized pigs, 16 were euthanized during lactation, 16 during the weaner stages, 8 during growing and 5 during finisher stage. These animals were selected for euthanasia on the basis of showing external lesions and/or pathologies such as hernias, severe tail biting (i.e., complete tail loss), severe lameness, external abscesses, emaciation, etc. Details on reasons for euthanasia and results for the study regarding respiratory pathologies will be presented in a separate manuscript.

CF pigs were at higher risk of death both during the pre-weaning period and during the entire production cycle (*P* < 0.05; Table 1). Similar odds ratios were observed for the risk of death in CFW1 and CFW2+; with both groups having a higher risk of death than NCF pigs (*P* < 0.05; Table 1).

**Table 1:** Odds ratios (OR) ±95% confidence intervals (CI) for the risk of mortality^1^ in 1,016 pigs followed from birth to slaughter in one commercial farm according to the lactation week when they were cross-fostered^2^.

	**Mortality from birth to weaning**			**Mortality from birth to slaughter**		
		**95% CI**			**95% CI**	
	**OR**	**Lower**	**Upper**	**OR**	**Lower**	**Upper**
NCF^3^ (*n* = 712) vs. CF^4^(*n* = 304)	3.31^a^	2.25	4.88	2.44^a^	1.72	3.37
NCF (*n* = 712) vs. CFW1^5^ (*n* = 124)	2.50^a^	1.42	4.26	2.00^a^	1.25	3.13
NCF (*n* = 712) vs. CFW2+^6^ (*n* = 180)	3.92^a^	2.53	4.76	2.78^a^	1.90	4.04
CFW1 (*n* = 124) vs. CFW2+ (*n* = 180)	1.56	0.88	2.85	1.39	0.83	2.34

1 *192 pigs died during the study. A total of 106 pigs died during the lactation period, 24 pigs died during the first and second weaner stages, 3 pigs died during growing, 14 pigs died during the finishing stage and 45 pigs were euthanized*.

2 *Pigs were retrospectively classified according to the week of lactation when they were CF*.

3 *NCF, non cross-fostered pigs*.

4 *CF, Cross-fostered pigs regardless of lactation week when CF occurred*.

5 *CFW1, Pigs cross-fostered during the first week of lactation*.

6 *CFW2+, Pigs cross-fostered during the second or third week of lactation*.

a *Different from reference category; P < 0.05*.

Using the nested case-control design, there were no observed differences in ADG between CF weeks (*P* > 0.05). There was an effect on BW (*P* < 0.05); on average CFW2+ pigs were ~2.5 kg lighter than NCF and 2.1 kg lighter than CFW1 pigs at each weigh-in; however, such differences disappeared (*P* > 0.05) once the Tukey adjustment was applied to the multiple comparisons. ADG and BW increased as time progressed irrespective of CF week (*P* < 0.05; Table 2). There was no interaction between CF week and production stage (*P* < 0.05).

**Table 2:** Average daily gain and body weight (least square mean ± standard error) at different stages of the production cycle for 124 pigs classified according to the lactation week when they were cross-fostered and selected for a nested case control design^1^.

	**Non cross-fostered (*n* = 62)**		**Cross-fostered in week 1 (*n* = 31)**		**Cross-fostered in week 2+ (*n* = 31)**		***P*-value^2^**
	**LS mean**	***SE***	**LS mean**	***SE***	**LS mean**	***SE***	
**AVERAGE DAILY GAIN, KG**							
Weaning	0.24^a^	0.02	0.23^a^	0.03	0.18^a^	0.03	0.233
Weaner stage 1	0.25^a^	0.02	0.25^a^	0.03	0.24^a^	0.03	
Weaner stage 2	0.68^a^	0.02	0.72^a^	0.03	0.70^a^	0.03	
Grower stage	1.06^a^	0.02	1.00^a^	0.03	1.00^a^	0.03	
**BODY WEIGHT, KG**							
Weaning	7.85^a^	0.73	7.24^a^	1.02	5.85^a^	1.02	0.323
Weaner stage 1	13.45^a^	0.74	12.85^a^	1.05	11.17^a^	1.04	
Weaner stage 2	31.95^a^	0.74	32.70^a^	1.08	30.35^a^	1.07	
Grower stage	67.81^a^	0.73	66.27^a^	1.02	63.28^a^	1.03	

1 *Pigs were matched by sow parity, birth weight and number of piglets born alive*.

2 *P value reported for the interaction between cross-fostering week and production stage*.

a *No statistical difference observed between cross-foster week, P > 0.05*.

**Figure 2:**
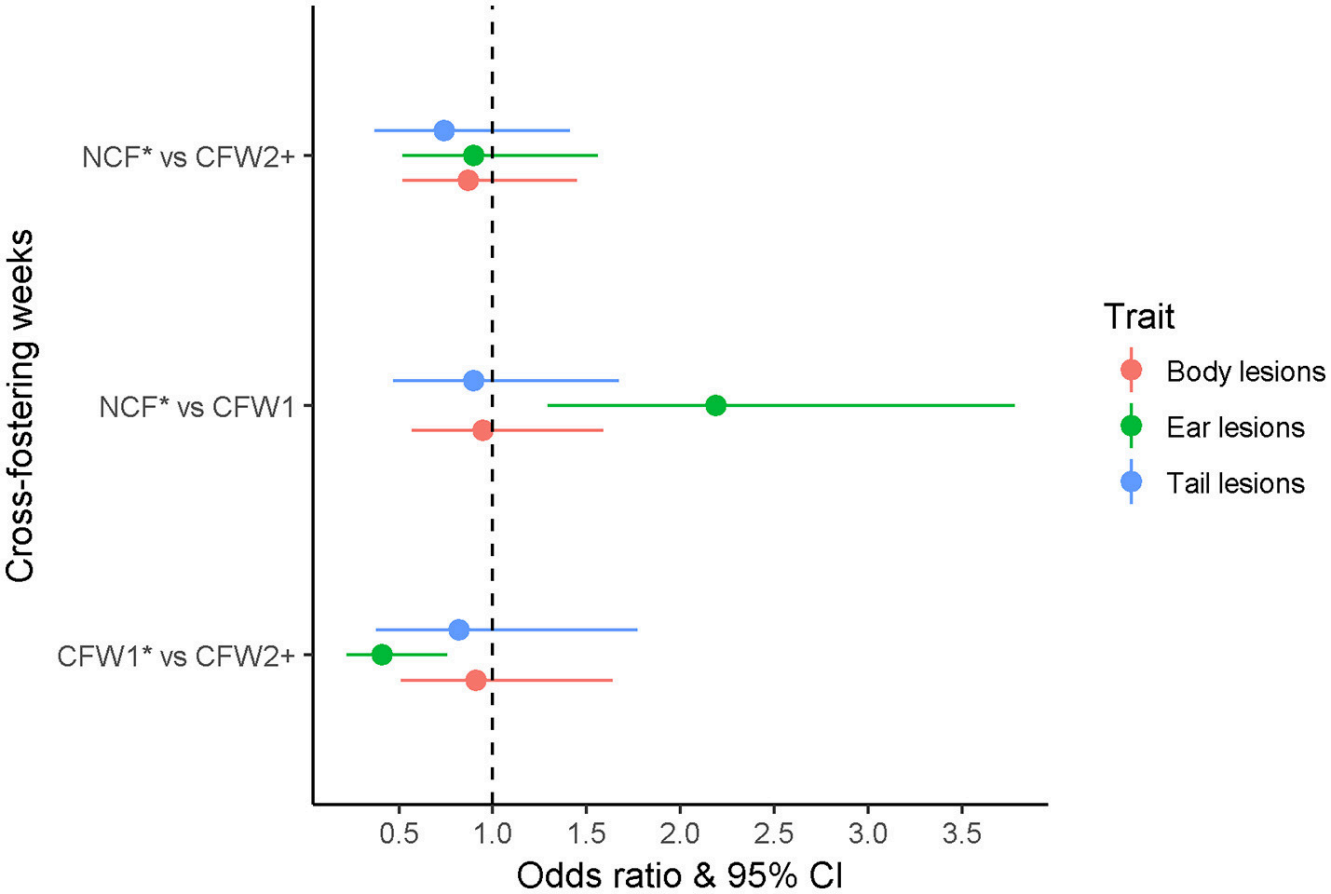
Odds ratios and 95% confidence interval (CI) for body, ear and tail lesions for 124 pigs classified according to the lactation week when they were cross-fostered as non-cross-fostered, cross-fostered during the first or second or third week of lactation. Pigs originated from one batch of 1,016 pigs born within 1 week that was followed from birth to slaughter in a farrow-to-finish commercial farm. Pigs were selected from each cross-fostering week in a nested case control study matched by parity, birth weight and number of litters born alive. ^*^NCF, Non cross-fostered; CFW1, cross-fostered during the first week of lactation; CFW2+, cross-fostered during the second or third week of lactation.

**Figure 3:**
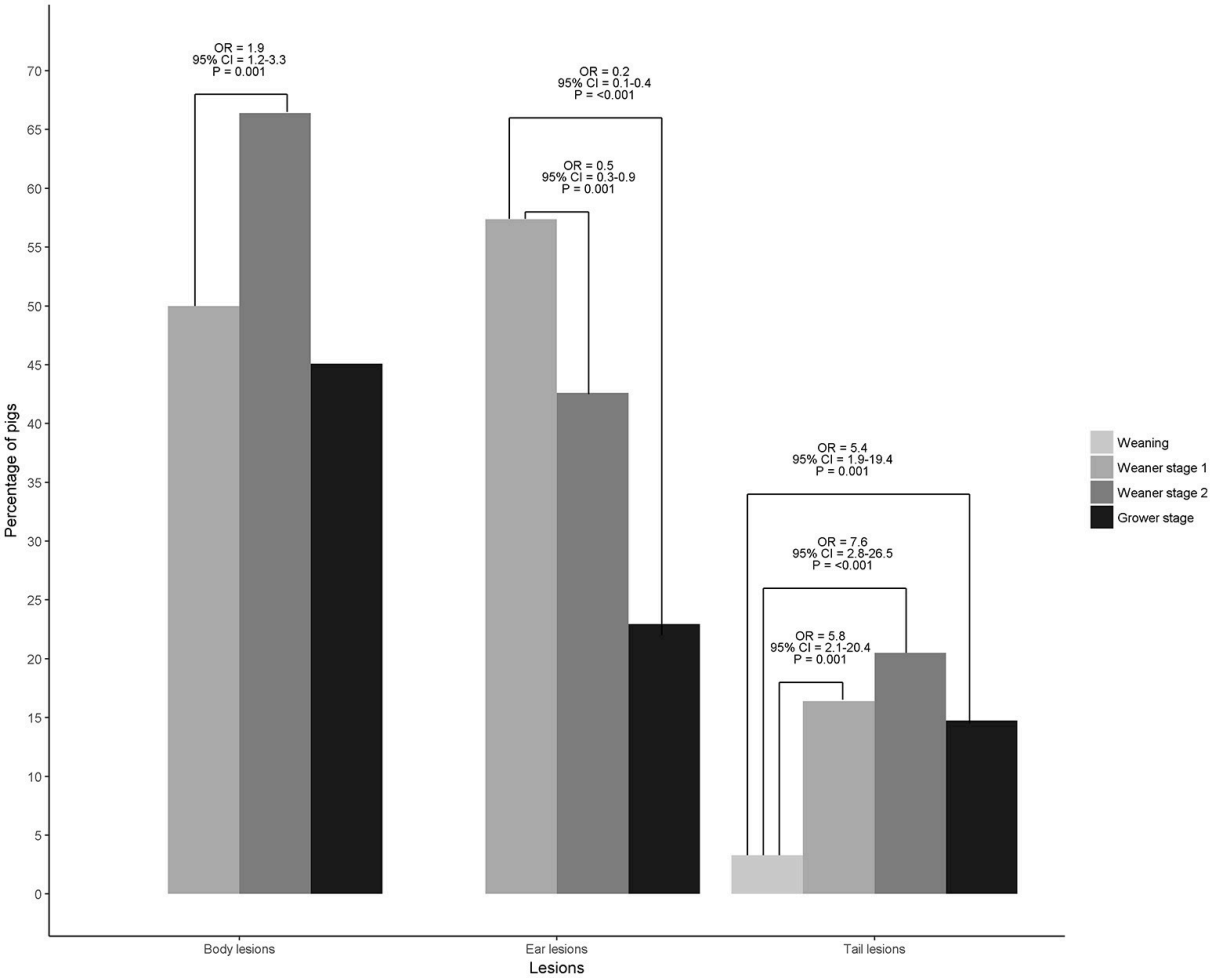
Percentage of pigs, odds ratios (OR) and 95% confidence interval (CI) for body, ear and tail lesions at each different production stage. The figure includes 124 finisher pigs from 1 batch of 1,016 pigs born within 1 week that was followed from birth to slaughter in a farrow-to-finish commercial farm. Pigs were selected from each cross-fostering week in a nested case control study matched by parity, birth weight and number of litters born alive. Body and ear lesions were not observed at weaning.

There were no observed differences between CF week for the presence of tail and body lesions (*P* > 0.05). There was no difference in the likelihood of ear lesions between NCF and CFW2+ (*P* > 0.05); however, CFW1 were more likely to have ear lesions than NCF and CFW2+ (*P* < 0.05; Figure 2). Pigs were more likely to have tail lesions at the end of the first and second weaner and grower stages compared with at weaning (*P* < 0.05). Similarly, pigs were more likely to have body lesions at the end of the second weaner compared with the first weaner stage (*P* < 0.05). Ear lesions were less likely to be present at the end of the second weaner and grower stages compared with at the end of the first weaner stage (*P* < 0.05; Figure 3).

### Welfare and health indicators at slaughter and carcass traits

CF week was not associated with any of the welfare and health indicators at slaughter (*P* > 0.05). Lower cold carcass weight was observed (*P* = 0.05) for CFW2+ pigs (84.8 ± 1.76 kg) compared with NCF (90.0 ± 1.28 kg) and CFW1 (89.4 ± 1.76 kg) pigs. Similarly, CFW2+ pigs had lower muscle depth (51.0 ± 0.69 mm) compared with NCF (53.4 ± 0.50 mm) and CFW1 (54.6 ± 0.69 mm) pigs. There was no difference between CF week in terms of lean meat % and fat thickness.

## Discussion

There is limited research about CF practices under commercial conditions and, to our knowledge, results from this study represent the first attempt to characterize CF practices in a farrow-to-finish commercial farm and its associations with pig performance, health and welfare traits in 20 years. Two CF practices were observed; piglets from larger litters were CF early in lactation and piglets with lower birth BW were CF late in lactation. Cross-fostering was associated with a higher risk of death during lactation as well as during the entire production cycle. Early CF was associated with a higher likelihood of having ear lesions and no differences were observed between CF weeks in the likelihood of having body or tail lesions. Even though performance did not differ between CF weeks, late CF was associated with lower carcass weight and less carcass muscle depth.

A large percentage (29.9%) of pigs were cross-fostered in the present study which is higher than the mean percentage of 8.6% of cross-fostered piglets reported by Straw et al. (1) for 96 farms in the USA and Canada. In the current study, pigs cross-fostered in the first week of lactation were likely moved to reduce variation in litter size as they were born to sows with larger litters (i.e., higher NBA) than NCF pigs. This management strategy is common practice on commercial farms and its purpose is to match sows' rearing capacity with litter size to ensure that all piglets can access a functional teat (3). Straw et al. (1) estimated that up to 5% of piglets should be cross-fostered to achieve this purpose. In this study, 12.2% of total NBA (i.e., 124 CFW1 pigs out of 1,016 pigs) were cross-fostered to standardize litter size. This is likely because genetic improvements in sow prolificacy have led to larger litter sizes and thus, a greater percentage of pigs needing to be moved to achieve uniformity of litter sizes. Nonetheless, this poses a management challenge as surplus piglets exceed sows' rearing capacity, hence other cross-fostering practices involving the use of nurse sows or artificial rearing systems should be considered (3).

Results suggest that CF during the second or third week of lactation was done to standardize BW within litters as these pigs were, on average, 0.14 kg lighter at birth than NCF pigs. It is likely that lighter pigs at birth continued to weigh less throughout the lactation period as lower birth BW is associated with reduced growth performance (9, 25). The main reason why attempts are made to standardize the size of piglets within a litter is to increase the competitive ability of the smaller piglets at the time of feeding independently of litter size (26). In this study, 17.7% of total NBA (i.e., 180 CFW2+ pigs out of 1,016 pigs) were cross-fostered to standardize BW within litters. This percentage falls in the range of 15–20% suggested by Straw et al. (1) to achieve this purpose.

One of the main purposes of cross-fostering is to reduce piglet pre-weaning mortality (2, 6, 27). However, an increased risk of mortality was observed in cross-fostered pigs both during the lactation period as well as during the whole production cycle and no difference in the risk of mortality was observed between CFW1 and CFW2+ pigs. The similar odds ratios for mortality during both time periods (i.e., lactation and the whole production cycle) are likely due to the fact that only 36.5% of pigs died during the weaner, grower, and finisher periods (vs. 63.5% of pigs that died during lactation) and thus, including those pigs in the statistical analysis doesn't influence the results dramatically. Furthermore, risk of mortality was similar between CFW1 and CFW2+ pigs which agrees with results reported by Straw et al. (1) when comparing limited (i.e., 2 days after farrowing) to late cross-fostering (i.e., >7 days after farrowing). Nonetheless, other underlying factors such as litter size and birth body weight, could contribute and thus, these results should be viewed with caution. For instance, CFW1 originated from litters with greater NBA and CFW2+ pigs were lighter at birth; and such parameters were previously identified as risk factors for piglet mortality (9). However, since this was not a controlled study and because larger litters and lower birth BW were confounded within CF week it is not possible to separate the risk of death due to the CF week and the aforementioned risk factors. Therefore, controlled studies should be carried out where piglets originating from large litters and piglets with low birth BW are randomly either NCF or cross-fostered in different lactation weeks.

Previous studies reported that limiting cross-fostering up to 2–3 days after farrowing has limited adverse effects on growth performance (2, 11) whereas cross-fostering 1 week after farrowing impaired weight gain (5, 12, 15). Nonetheless, cross-fostering is also associated with parameters such as sow parity, NBA and birth BW (9) all of which are also associated with growth performance (9, 25, 28). Differences in these parameters were found between cross-fostering weeks. Since we were interested in determining whether there was an association between cross-fostering week *per se* and performance, welfare and health, the nested case-control design allowed us to match pigs by sow parity, NBA and birth BW to investigate such associations independent of the other underlying factors.

On the basis of the nested case control analysis, there was no difference between cross-fostering weeks and ADG or BW; nonetheless, a numerical difference was observed. For instance, CFW2+ pigs weighed 25.5% less than NCF pigs and 19.7% less than CFW1 pigs at weaning. These results are in agreement with (5, 12, 15) for piglets cross-fostered 1 week after farrowing. Moreover, such differences in BW, albeit smaller, persisted throughout the production cycle. In fact, carcasses from CFW2+ pigs were 4.9 kg lighter and also had less muscle depth (the biological significance of which is questionable as the difference was only 3 mm). Similar results were previously reported by Powell and Aberle (29) in runts (i.e., the smallest piglets in the litter). The authors reported that *Longissimus* muscle area was 5.8 cm^2^ greater and estimated carcass muscle % was 4.2% higher in NCF runts compared with CF runts. Therefore, further research is necessary to understand possible implications of cross-fostering on carcass quality traits.

Late cross-fostering increases fights (5, 13) during suckling as pigs compete to gain access to a specific teat and Robert and Martineau (5) observed a high number of body and face lesions in cross-fostered pigs at days 1, 7, 13, and 16 of lactation. Body lesions were not observed at weaning in any of the studied pigs. Fights in young piglets (i.e., <20 days of age) are less than 5 min long and lesions are relatively mild (30). Thus, it is probable that any lesion resulting from fighting had already healed at weaning. Additionally, since most fighting during lactation between NCF and CF piglets occurs during suckling (5), the lack of body lesions at weaning could be interpreted as a sign of conflict resolution once the new teat order was established. No difference in the likelihood of having body lesions was observed between CF weeks during subsequent production stages. However, this does not mean that fighting did not occur or that body lesions were not present during the production cycle. Group composition changed on transfer to each production stage and the percentage of pigs with body lesions reflected such changes. For instance, as shown in Figure 3, 50% of pigs had skin lesions regardless of CF week at the end of the first weaner stage and this percentage increased to 66% at the end of the second weaner stage and decreased to 45% at the end of the grower stage. At weaning, pigs originating from 4 to 5 entire litters [see (9)] were housed together during the first weaner stage in groups of c. 55 pigs. On transfer to the second weaner stage, groups were split and groups of c. 36 pigs were formed. It is likely pigs fought to establish a new dominance hierarchy and hence the increase in the percentage of pigs with body lesions. Pigs remained in the same groups during the grower stage, thus there was no need to establish a new dominance hierarchy and the percentage of pigs with body lesions decreased as some lesions likely healed.

Tail lesions are an indication that pigs cannot cope within their environment (31). There are several factors affecting the occurrence of tail lesions including genetics (32), sex (33), weaning age, flooring type, provision of enrichment, fighting, stress and stocking density (8, 31, 33, 34). Moinard et al. (8) found a higher incidence of tail lesions on farms where CF was practiced; though, whether the presence of tail lesions was higher in CF or NCF is unclear. In this study, no differences in the presence of tail lesions were observed between CF weeks. However, tail lesions were observed throughout the entire production cycle and similar to previous findings, presence of tail lesions increased as time progressed (35–37). Based on our records, it is not possible to ascertain a specific reason for this but a possible explanation could be that similar to body lesions the increase in tail lesions was related to re-mixing of pigs between the production stages. Mixing of unfamiliar pigs disrupts social group stability increasing stress levels (38). Stress can contribute to tail biting behavior (34) although it is unknown if stress predisposes a pig to become an initiator or a recipient of such abnormal behavior.

Ear lesions are becoming increasingly common in intensive pig farms (31). Their etiology and risk factors are not fully understood; however, it is possible that similar risk factors are shared between ear and tail lesions. In contrast to tail lesions, we did observe differences in the presence of ear lesions between CF weeks. Pigs in this study shared the same genetics, rearing conditions and were of similar age. Thus, it is logical to assume that they experienced the same risk factors for ear lesions. Ear lesions were present in all the stages of the weaner-grow period which agrees with previous reports (37, 39); but in contrast to tail lesions, they decreased as pigs progressed through the production stages. Ear and tail lesions are multifactorial in nature. It is possible that even if they share risk factors, different combinations of risk factors only pose a risk for ear and tail lesions at certain time points. However, there are few studies investigating risk factors for ear lesions and thus, this warrants further investigation. However, we speculate that as pigs get older they can more actively and aggressively defend their ears from attention by others. This may cause biting pigs to switch attention away from the head and toward the more easily accessible tail. Furthermore, competition for access to feed may intensify as pigs get older and heavier (with an associated reduction in space allowance at the trough) thereby making attacks to the rear of the pig more likely.

The greater risk of ear lesions for CFW1 compared with CFW2+ pigs could partially be explained by differences in BW as an increased risk of ear lesions is associated with heavier BW (39). However, this does not explain the greater risk of ear lesions in CFW1 compared to NCF pigs as their BW was similar. In light of the lack of information regarding possible risk factors for ear lesions, and the fact that pigs were subjected to the same risk factors, arguably our results indicate that cross-fostering within the first week of life indirectly pre-disposes pigs to ear lesions. We postulate that this could be mediated by stresses inherent to cross-fostering which include separation from their own mother, handling, re-mixing with unfamiliar piglets and the associated fighting. Severe stress on piglets in the perinatal period makes them more stress-susceptible later in life (40, 41). Hence it is possible that cross-fostering when the piglets were very young represented such a severe stress that their immune function was compromised to the extent that they were more predisposed to ear lesions. However, CFW2+ pigs were also severely stressed given their lower body and carcass weight and yet they were not at increased risk of ear lesions. The difference may lie in the critical timing of the developmental stage of the pigs at the point of cross-fostering and it may be that stress related with early cross-fostering combined with stresses associated with faster growth rate in the CFW1 pigs acted to increase their susceptibility to ear lesions compared to both NCF and CFW2+ pigs.

We suggest that cross-fostering should be minimized and then, based on the results of this study, and in line with previous recommendations (4), only early cross-fostering should be applied. Early cross-fostering was performed on piglets coming from larger litters with similar birth weights as non-cross fostered pigs and no negative effects on carcass yield were observed. Late cross-fostering was performed in low birth weight piglets; however, such small piglets are likely to continue to grow at a slower rate (42–44). It is unclear what role early cross-fostering plays in the development of ear lesions; however, our results suggest that there is some association, albeit likely an indirect link. If the higher presence of ear lesions in early cross fostered piglets is mediated by poorer immune development, cross-fostering such piglets to sows that are at the same stage of lactation could help to ensure adequate colostrum and milk intake such that immune development is not compromised.

## Conclusion

Different cross-fostering practices were observed with early cross-fostering apparently aimed at reducing variation in litter size to ensure piglets can access functional teats and with late cross-fostering apparently aimed at reducing BW variation within litters. Although it could be argued that these cross-fostering strategies were particular to the farm on which the study was conducted it is likely that similar strategies are in place on most commercial farms trying to deal with the challenges posed by large litters of piglets with highly variable body weights. Performance and health traits were similar between CF weeks but early cross-fostering could have an indirect negative impact on ear lesions by inducing stress and making them more prone to ear lesions. However, the greater risk of ear lesions observed in early cross-fostered pigs remains unexplained and controlled studies that include behavioral observations as well as immunological measures should be conducted to try to understand the role cross-fostering plays in the development of ear lesions. Unfortunately, we are unable to provide a precise explanation for the decrease in ear lesions while tail lesions increase. However, these results are similar to those previously reported where an increase in one of the lesions is met with a decrease in the other lesion or vice versa (35, 37). Clearly further research on the etiology of ear and tail lesion development and the dynamics of both are needed.

Results regarding the observed higher risk of mortality in cross-fostered piglets should be viewed with caution as other underlying factors such as sow parity, litter size and birth body weight likely also contributed to the results. However, due to the observational nature of this study, we were unable to separate the risk of death due to CF week and the aforementioned risk factors. Controlled studies should be carried out where piglets originating from large litters and piglets with low birth BW are randomly either NCF or cross-fostered in different lactation weeks.

## Ethics statement

The study received ethical approval from the Teagasc Animal Ethics Committee (TAEC 40/2013).

## Author contributions

JC and EG: data collection, data management, data analysis, drafting of manuscript; AD: data collection and drafting of manuscript; LB: study coordinator, data collection, drafting of manuscript. All authors read and approved the final manuscript.

### Conflict of interest statement

The authors declare that the research was conducted in the absence of any commercial or financial relationships that could be construed as a potential conflict of interest.
